# Impact of in-hospital COVID-19 quarantine policy changes on quality of acute stroke care: a single center experience

**DOI:** 10.3389/fneur.2024.1488529

**Published:** 2025-01-07

**Authors:** Minkyung Kim, Keon-Joo Lee, Seong-Eun Kim, Hokyu Kim, Jung Hoon Han, Han Jun Kim, Kyungmi Oh, Sung-Jun Park, Chi Kyung Kim, Young-Duck Cho

**Affiliations:** ^1^Department of Neurology, Korea University Guro Hospital, Korea University College of Medicine, Seoul, Republic of Korea; ^2^Department of Neurology and Cerebrovascular Center, Seoul National University Bundang Hospital, Seoul National University College of Medicine, Seongnam, Republic of Korea; ^3^Department of Emergency Medicine, Korea University Guro Hospital, Korea University College of Medicine, Seoul, Republic of Korea

**Keywords:** COVID-19, stroke, ischemic stroke, quality of care, quarantine

## Abstract

**Introduction:**

The COVID-19 pandemic is known to impact in-hospital processes for acute stroke patients, potentially resulting in delays due to quarantine and screening measures. The purpose of this study was to determine effects of changes in in-hospital quarantine policies on quality of care for acute stroke patients.

**Methods:**

Hyperacute ischemic stroke patients who were admitted to Korea University Guro Hospital between January 2019 and February 2021 via the emergency department were included in this study. All had neurological symptoms within 6 h before arrival. As a mandatory COVID-19 real-time PCR screening test was implemented in March 2020, changes in quality indicators according to the progress of COVID-19 pandemic and changes in in-hospital quarantine policy, including door-to-image time (DIT), door-to-referral time, door-to-needle time (DNT), door-to-puncture time (DPT), and functional outcomes (discharge and 3-month modified Rankin’s scale) were determined.

**Results:**

A total of 268 hyperacute stroke patients were analyzed. The number of hyperacute stroke patients gradually decreased as the pandemic progressed. Time indicators, including door-to-referral time, DIT, and DPT during the pandemic were increased. When pre-and post-COVID-19 screening epochs were compared, DIT, door-to-neurologist referral time, and DPT showed numerical increases. However, after accounting for potential confounders, a significant delay in DIT was found to be associated with the in-hospital COVID-19 quarantine policy.

**Discussion:**

Our study showed that enhancing in-hospital COVID-19 quarantine measures might increase the response time for hyperacute stroke care, suggesting an impact on the quality of care.

## 1 Introduction

As well emphasized in the famous catchphrase “time is brain,” rapid diagnosis and quick achievement of reperfusion are crucial for hyperacute stroke management to minimize brain injury. Thus, time indices such as door-to-imaging time (DIT), door-to-needle time (DNT), and door-to-puncture time (DPT) are widely acknowledged as quality indicators of stroke care. For example, the Get With The Guidelines (GWTG)-Stroke program has proposed the following targets to reach: door-to-imaging time within 25 min, door-to-needle time within 60 min, and door-to-puncture time within 2 h (1–3).

Starting from the year 2019, the COVID-19 pandemic has brought worldwide chaos and significantly impacted global lifestyle, including the healthcare system (4). Emergency care system, including that for acute ischemic stroke, is not an exception (5). The pandemic has resulted in delays in the time course of reaching to treatment for acute stroke patients, such as elongated time from symptom detection to hospital arrival in the community, leading to worse functional prognosis (6, 7). After the first COVID-19 case in Korea, the Korean government has implemented quarantine policies, requiring suspected COVID-19 patients to be isolated at home and confirmed cases to be placed in residential treatment centers. In addition, each hospital has implemented its quarantine policies based on circumstances (8, 9).

This study aimed to determine changes in time indices of acute stroke care during the COVID-19 pandemic and effects of in-hospital quarantine policies on these time indices within a single medical center.

## 2 Methods

### 2.1 Data collection

This retrospective observational study was conducted at Korea University Guro Hospital. Patients aged 18 years or older who visited the emergency department between January 1st, 2019 and February 19th, 2021 with acute stroke symptoms presented within 6 h and final diagnosis of ischemic stroke were included. Demographic information (including age and sex), premorbid modified Rankin Scale (mRS), stroke risk factors, comorbidities, and initial National Institute of Health Stroke Scale (NIHSS) scores were collected for all patients during hospitalization upon arrival. This study was approved by the ethics committee of Korea University Guro Hospital (IRB No. 2024GR0006).

Information for stroke risk factors and comorbidities included smoking history and the presence of hypertension, diabetes mellitus, hyperlipidemia, atrial fibrillation, cancer, coronary heart disease, or previous history of stroke or transient ischemic attacks (TIA). Cancer status was determined based on whether patients were currently undergoing cancer treatment or had been diagnosed with cancer within the past 5 years. Coronary heart disease included a history of angina or myocardial infarction. It was determined based on whether patients were undergoing percutaneous coronary intervention or had coronary artery stenosis exceeding 50% of the arterial diameter on coronary angiography or CT scan. Stroke subtypes were classified using the TOAST classification determined by the attending stroke physician (10). Initial brain images (CT or MR angiography) were retrospectively reviewed and large artery occlusion of the cerebral arteries was determined if there was an occlusion in a large intra or extracranial artery (M1 or proximal M2 segment of middle cerebral artery, A1 segment of anterior cerebral artery, P1 segment of posterior cerebral artery, intracranial or extracranial internal carotid artery, basilar artery and vertebral artery) relevant to the infarct lesion. Time indices, including onset-to-arrival time, door-to-neurologist referral time, DIT, DNT, DPT, and mRS scores measured at discharge and 3 months, were used as quality indicators (2, 6, 11–13). Onset-to-arrival time was defined as difference between the time of the first symptom onset and the time arriving at the emergency department. Door-to-neurologist referral time was defined as the time when the emergency clinician referred the patient to a neurologist after their arrival. DIT was the duration between the patient’s arrival and the acquisition of brain imaging such as brain CT or MRI. DNT and DPT represented the time taken to initiate intravenous thrombolysis and endovascular thrombectomy, respectively.

### 2.2 Changes in in-hospital COVID-19 quarantine policy

Before the COVID-19 pandemic, we had established a fast tract system for prompt diagnosis and managing hyperacute stroke patients who visited the emergency department. Acute stroke symptoms encompassed neurological deficits such as dysarthria, aphasia, unilateral limb weakness, or mental changes. If patients were initially presented with these symptoms at the emergency department, then a fast-tract protocol was activated, involving immediate direct contact with the neurologist and acquisition of brain image (CT or MRI). Intravenous thrombolysis or endovascular thrombectomy was also performed if needed.

All patients visiting the emergency department after January 20th, 2020, the date when the first case of COVID-19 was confirmed in Korea, underwent a survey to determine whether they had recently visited China, had encountered a confirmed case of COVID-19, or had exhibited COVID-19 symptoms. Subsequently, all patients who visited the emergency department underwent chest X-rays to screen for pneumonia, and only those with suspicious pneumonia underwent RT-PCR testing for COVID-19. The RT-PCR tests were conducted using samples collected from the nasal and throat swabs. Such tests took approximately 1 h to yield results. Only after ruling out the possibility of COVID-19 infection were patients permitted to undergo endovascular thrombectomy or be admitted to the stroke unit. As the COVID-19 pandemic worsened (Supplementary Figure S1), every patient who visited the emergency department underwent COVID-19 screening regardless of chest X-ray results and were allowed to proceed for endovascular thrombectomy or hospital admission only if they got negative results in accordance with the in-hospital quarantine policy change on March 20th, 2020. Since patients presenting with acute stroke symptoms were potential candidates for intervention or admission, the COVID-19 test by taking a nasal swab before proceeding to brain images became a routine process for all patients.

### 2.3 Statistical analysis

Baseline characteristics of study subjects are described by mean and standard deviation (SD) for interval variables, median and interquartile range (IQR) for ordinal variables, and frequencies with proportions for categorical variables. Number of patients, number of reperfusion therapies (intravenous thrombolysis and endovascular thrombectomy), and quality indicators (including time indexes and clinical outcomes) are described according to each quarter of the year. mRS score at discharge and 3 months were dichotomized into 0–2 vs. 3–6, with mRS of 0–2 being an indicator of good functional outcome. Nine patients lacked 3-month mRS scores. Thus, the analysis for the 3-month mRS was performed as a complete-case analysis exclusively for those with such information. Crude trend of quality indicators according to calendar date was evaluated using Spearman’s rank correlation test and chi-square test for trend, and thereafter, quality indicators were compared using the Mann–Whitney *U* test between before and after the in-hospital quarantine policy change on March 20th, 2020. For multivariable analysis, calendar date of arrival to the emergency department was implemented into the model as continuous variables and quality indicators were log-transformed. Multivariable analysis was conducted to determine independent effects of calendar date on quality indicators by employing the following sets of covariates to the linear regression model: initially without any other covariates for Model 1, incorporating age, sex, premorbid mRS, initial NIHSS, and onset-to-arrival time for Model 2, and encompassing all other covariates (age, sex, premorbid mRS, initial NIHSS, onset-to-arrival time, hypertension, diabetes, dyslipidemia, atrial fibrillation, malignancy, smoking, history of ischemic heart disease, history of stroke or TIA, and stroke subtype determined by the TOAST classification) for Model 3. Additionally, to explore the effect of the in-hospital quarantine policy change, the variable with information of whether the patient arrived before or after the in-hospital quarantine policy change was implemented in each model. All statistical analyses were carried out using the R software version 3.3.0+ (R Foundation for Statistical Computing, Vienna, Austria). A threshold for statistical significance was set at *p* < 0.05.

## 3 Results

Between January 1st, 2019 and February 19th, 2020, a total of 268 individuals who visited the emergency department presented with stroke symptoms within 6 h after a final diagnosis of ischemic stroke. Among these patients, about two-thirds were males. The mean age was 69 years old. Their initial National Institutes of Health Stroke Scale (NIHSS) score upon admission was 5 (IQR: 3–12). A significant proportion of patients had a history of hypertension, accounting for more than half of cases (61%), while over 25% of patients were diagnosed with diabetes mellitus. Notably, 49 (18.3%) patients had a prior medical history of stroke or transient ischemic attack. Among stroke subtypes, large artery atherosclerosis accounted for the highest at approximately one-third, followed by cardioembolism (26.9%) and small vessel occlusion (19.0%). Ninety-five (35%) patients had a large artery occlusion relevant to the infarct lesion. In terms of treatment, about one-third and 20% received intravenous thrombolysis and endovascular thrombectomy, respectively (Table 1).

**Table 1:** Baseline characteristics of the study population (*N* = 268).

Variables	Value
Age, mean ± SD	68.6 ± 12.3
Sex, male (%)	179 (66.8)
Premorbid mRS, median (IQR)	0 (0–0)
Initial NIHSS score, median (IQR)	5 (3–12)
Comorbidities, *N* (%)	
Hypertension	162 (60.5)
Diabetes	77 (28.7)
Hyperlipidemia	33 (12.3)
Atrial fibrillation	34 (12.7)
Cancer	28 (10.5)
Smoking	39 (14.6)
Coronary heart disease	19 (7.1)
Stroke or TIA	49 (18.3)
Stroke subtype, *N* (%)	
Large artery atherosclerosis	84 (31.3)
Small vessel occlusion	51 (19.0)
Cardioembolism	72 (26.9)
Other-determined	15 (5.6)
Undetermined	46 (17.2)
Large artery occlusion, *N* (%)	95 (35.4)
Hyperacute reperfusion treatment, *N* (%)	
Intravenous thrombolysis	93 (34.7)
Endovascular thrombectomy	54 (20.2)

SD, standard deviation; mRS, modified Rankin’s Scale; NIHSS, National Institute of Health Stroke Scale; IQR, interquartile range; TIA, transient ischemia attack.

**Figure 1:**
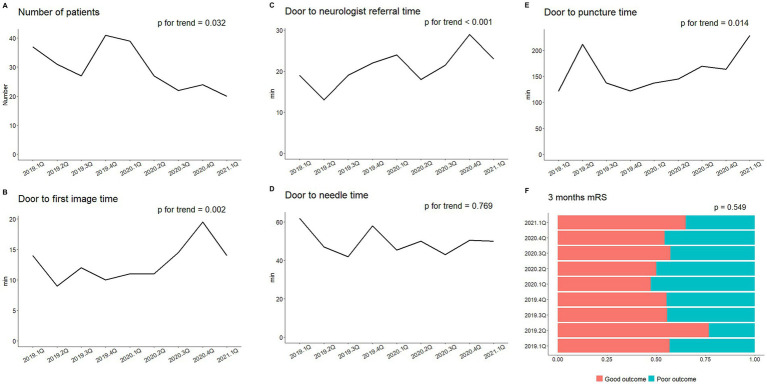
Impact of COVID-19 pandemic on quality indicators for hyperacute stroke care. **(A)** Number of hyperacute stroke patients who visited the emergency department. **(B)** Door-to-imaging time. **(C)** Door to neurologist referral time. **(D)** Door to needle time. **(E)** Door-to-puncture time. **(F)** Three-month modified Rankin’s Scale (mRS) dichotomized into two groups: “good outcome” (mRS = 0–1) and “poor outcome” (mRS > 2).

When looking into the trend between each quarter of the year and quality indicators, we observed a gradual decrease in the number of patients with a concomitant increase in new COVID-19 cases in the community over time (Figure 1A and Supplementary Table S1). Additionally, increasing trends were noted for door-to-neurologist referral time, DIT, and DPT. However, other quality indicators such as DNT and discharge or 3-month mRS exhibited no differences (Figures 1B–F and Supplementary Table S1).

In the multivariable analysis to determine effects of calendar date on quality indicators (Table 2), calendar date seemed to increase DIT and door-to-neurology-referral time in Model 2 after adjusting for age, sex, premorbid mRS, initial NIHSS, and onset-to-arrival time. Furthermore, DPT seemed to be increased after incorporating other covariates (Model 3).

**Table 2:** Multivariable analysis for effects of calendar date (per 30 days) on quality indicators.

Quality indicator	Standardized beta [95% CI]	*p*-value
Model 1		
Door to first image time	0.193 [0.074–0.311]	0.002
Door to neurologist referral time	0.195 [0.077–0.313]	0.001
Door to needle time	−0.047 [−0.256–0.162]	0.656
Door to puncture time	0.289 [0.022–0.555]	0.034
Discharge mRS 0–2	0.0002 [−0.0010–0.0014]	0.750
3-month mRS 0–2	0.0004 [−0.0008–0.0015]	0.543
Model 2		
Door to first image time	0.178 [0.064–0.292]	0.002
Door to neurologist referral time	0.195 [0.078–0.313]	0.001
Door to needle time	−0.061 [−0.275–0.153]	0.575
Door to puncture time	0.279 [−0.017–0.575]	0.064
Discharge mRS 0–2	−0.0004 [−0.0019–0.0010]	0.565
3-month mRS 0–2	0.0003 [−0.0011–0.0018]	0.665
Model 3		
Door to first image time	0.201 [0.085–0.316]	<0.001
Door to neurologist referral time	0.203 [0.084–0.323]	<0.001
Door to needle time	−0.082 [−0.300–0.136]	0.456
Door to puncture time	0.538 [0.187–0.890]	0.004
Discharge mRS 0–2	−0.0002 [−0.0018–0.0014]	0.814
3-month mRS 0–2	0.0004 [−0.0012–0.0020]	0.604

Model 1: unadjusted for covariates. Model 2: adjusted for covariates: age, sex, premorbid mRS, visit NIHSS, and onset to arrival time. Model 3: adjusted for covariates such as age, sex, pre mRS, visit NIHSS, onset to arrival time, history of hypertension, diabetes, dyslipidemia, atrial fibrillation, cancer, smoking, ischemic heart disease, previous stroke or transient ischemia attack, and stroke subtype.

After that, we divided patients into those who arrived in our emergency department before (*n* = 173) and after (*n* = 95) the change in in-hospital quarantine policy with mandatory COVID-19 screening. There were no differences in baseline characteristics between these groups except that the premorbid mRS was slightly higher after implementation of the mandatory COVID-19 screening (Supplementary Table S2). However, the proportion of patients treated with endovascular thrombectomy was much higher after the change in in-hospital quarantine policy (15.6% before mandatory COVID-19 screening vs. 28.4% after the mandatory COVID-19 screening). A delay in median DIT was observed comparing before and after the change in quarantine policy (11 min vs. 14 min). Although it did not reach the statistical significance threshold, median door-to-referral time (20 min vs. 23 min) and median DPT (137 min vs. 151.5 min) were also prolonged after the change in the mandatory COVID-19 screening policy. Despite these shifts in quality indicators, no substantial differences were noted in functional outcomes such as discharge mRS scores or 3 months’ mRS scores (Table 3).

**Table 3:** Comparison of quality indicators before and after quarantine in-hospital quarantine policy change.

Quality indicator	Before mandatory COVID-19 screening (*N* = 173)	After mandatory COVID-19 screening (*N* = 95)	*p-*value
Door to first image time, minutes [median (IQR)]	11 (7–18)	14 (10–24)	< 0.01
Door to neurologist referral time, minutes [median (IQR)]	20 (13–29)	23 (16–35)	0.08
Door to needle time, minutes [median (IQR)]	51 (39–59)	50 (45–58)	0.70
Door to puncture time, minutes [median (IQR)]	137 (120–170)	158 (133–192)	0.09
Discharge mRS, 0 to 2 (%)	65 (37.6)	39 (41.1)	0.67
3-months mRS, 0 to 2 (%)	96 (57.5)	52 (56.5)	0.99

mRS, modified Rankin’s Scale; IQR, interquartile range.

After introducing information of whether the patient was admitted before or after implementing the mandatory COVID-19 screening as a variable in addition to previous multivariable models, the change in the quarantine policy seemed to increase the DIT even after adjusting for other covariates (Table 4).

**Table 4:** Multivariable analysis for effects of changes of in-hospital quarantine policy on quality indicators.

Quality indicator	Estimate [95% CI]	*p*-value
Model 1		
Door to first image time	0.410 [0.019–0.800]	0.040
Door to neurologist referral time	0.008 [−0.386–0.402]	0.968
Door to needle time	0.417 [−0.238–1.072]	0.209
Door to puncture time	0.225 [−0.523–0.973]	0.549
Discharge mRS 0–2	−0.547 [−1.398–0.279]	0.199
3-month mRS 0–2	−0.217 [−1.042–0.605]	0.604
Model 2		
Door to first image time	0.404 [0.028–0.779]	0.035
Door to neurologist referral time	0.024 [−0.367–0.415]	0.903
Door to needle time	0.435 [−0.241–1.111]	0.204
Door to puncture time	0.254 [−0.5382–1.047]	0.521
Discharge mRS 0–2	−0.605 [−1.664–0.427]	0.254
3 months mRS 0–2	0.024 [−0.978–1.029]	0.963
Model 3		
Door to first image time	0.399 [0.018–0.780]	0.040
Door to neurologist referral time	−0.052 [−0.450–0.346]	0.797
Door to needle time	0.249 [−0.479–0.976]	0.498
Door to puncture time	0.062 [−0.825–0.950]	0.888
Discharge mRS 0–2	−0.701 [−1.840–0.405]	0.219
3-month mRS 0–2	−0.150 [−1.216–0.913]	0.781

Model 1: adjusted for calendar date. Model 2: adjusted for covariates (age, sex, premorbid mRS, visit NIHSS, onset to arrival time, and calendar date). Model 3: adjusted for covariates such as age, sex, pre mRS, visit NIHSS, onset to arrival time, history of hypertension, diabetes, dyslipidemia, atrial fibrillation, cancer, smoking, ischemic heart disease, previous stroke or transient ischemia attack, stroke subtype, and calendar date.

## 4 Discussion

Our results indicate that there are challenges when managing patients who present with acute ischemic stroke during the COVID-19 pandemic. With the advent of the COVID-19 outbreak, there was a progressive decline in the number of patients presenting with acute stroke symptoms. Moreover, during the COVID-19 era, delays of time-to-neurology referral, DIT, and DPT were observed. Remarkably, implementation of the mandatory COVID-19 screening process for all patients during this period contributed to an increase of DIT, indicating its impact on intervention decision-making. While crucial for infection control, this policy notably disrupted timely management for acute stroke patients.

During the pandemic, cases confirmed with COVID-19 in Korea were required to be isolated in negative-pressure rooms within healthcare facilities or living treatment centers based on the severity of their condition (14). Additionally, those COVID-19 patients in Korea tended to avoid seeking healthcare services, although their situations needed such services, potentially having adverse effects on public health (15). This phenomenon was recognized globally. For example, one study has underscored how COVID-19 screening can disrupt optimal care including hospital admissions for cancer patients (16). Regarding stroke patients, a study conducted in China reported a decrease in the number of acute stroke patients visiting the emergency department after the onset of COVID-19, along with an observed increase in both door-to-onset time and door-to-needle time (17). Consistently, a study by Hsiao et al. (18) highlighted a decrease not only in acute stroke consultations but also in reperfusion treatment rates, emphasizing the need for education to ensure that patients in the community can access emergency care. Similarly, a meta-analysis has shown that the onset-to-arrival time of stroke patients is increased during the COVID-19 era because of a tendency to avoid hospital visits (19). Likewise, our study showed that the number of acute stroke patients decreased as the pandemic went on, which could be attributed to reluctance of patients to seek hospital care. The previously mentioned meta-analysis also highlighted that stroke response time was delayed within hospitals due to precautions such as symptom screening and additional isolation policies (19). Strict in-hospital isolation policies can also impact the management of acute ischemic stroke, leading to increased severity and in-hospital mortality rates (5). These not only affects acute stroke patients, but also has repercussions on general stroke patient care, including response times, treatment interventions, and stroke prevention, all of which are deteriorated after the onset of COVID-19 (19–21). These findings emphasize the need for a cautious approach when settling a policy regarding infection control to ensure it does not disrupt the process for acute stroke care.

In response to the advent of the COVID-19 pandemic, the “protected code stroke” was proposed as an approach to managing hyperacute stroke patients during the pandemic. This protocol included a simple screening questionnaire. If COVID-19 was suspected, personal protective equipment should be used when managing patients (22). The Korean Stroke Society has also issued a scientific statement noting that all medical staff should use personal protective equipment, minimize close contact with patients and in-hospital patient transportation, and limit advanced neuroimaging until COVID-19 is ruled out. However, it did not specify that COVID-19 must be excluded before procedures (23). During early stages of the COVID-19 pandemic, real-time RT-PCR assay was considered the gold standard for COVID-19 diagnosis. Although RT-PCR is known for its high sensitivity and specificity, it involves complex procedures and typically takes at least 4 h to get results, potentially causing delays in in-hospital processes (24–26). In response to these concerns, rapid antigen detection tests and rapid molecular assays were introduced. While these tests had somewhat lower sensitivity and specificity than RT-PCR, they were deemed suitable for certain criteria and eventually replaced RT-PCR (27, 28). The implementation of these new diagnostic tools has led to reduced emergency department stays and more efficient management of oncology patients (16, 29). Compared to RT-PCR, they are more cost-effective for acute management of trauma patients (26). Altogether, they have been proven to be valuable for improving management and enabling swift decision-making, although these rapid detection methods show lower sensitivity and specificity than RT-PCR. However, our center introduced RT-PCR as a screening tool, resulting in delays in acute stroke care. Therefore, cautious consideration regarding the necessity of confirmatory tests in emergent situations is essential as other infectious diseases may emerge in the future.

Our study focused on the effect of implementing an in-hospital quarantine policy on quality indicators in addition to worsening of the COVID-19 pandemic itself. Although door-to-neurologist referral time and door-to-puncture time showed delays, these results showed no significant differences after adjusting for potential confounders. Such results might be due to a low statistical power caused by a small sample size. However, implementation of the mandatory COVID-19 screening was found to be associated with a delay in DIT even after adjusting for potential confounders including the calendar date which accounted for worsening of the pandemic itself. In acute stroke patients, shortening the time from symptom onset to reperfusion therapy is the most critical factor affecting their prognosis (1, 30, 31). DIT is a crucial component of door-to-reperfusion time, signifying its central role in acute stroke management and patient outcomes (32). Some previous studies have shown that prolonged DIT can lead to delays in onset-to-treatment time, which in turn may impact a patient’s prognosis, although direct correlations between DIT and patient outcomes were not established in those studies (2, 33, 34). Both the National Institute of Neurological Disorders and Stroke guidelines and the American Heart Association/American Stroke Association recommend maintaining a DIT within 25 min to effectively minimize door-to-reperfusion time (2, 13, 35). This underscores the pivotal nature of door-to-imaging time in optimizing stroke management and improving patient outcomes (2, 13, 35). Several studies demonstrated significant efforts to reduce door-to-needle time and reperfusion time in the care of acute stroke patients, achieving meaningful reductions. However, even these studies consistently reported delays in door-to-image time despite these improvements (36, 37). Divergent from prior research studies that have primarily explored the effect of COVID-19 on acute stroke management, our study distinctly focused on how stringent quarantine policies could influence the quality of care for acute stroke patients. This emphasizes the significance of careful consideration before modifying quarantine policies for situations in which time is a crucial component of efficient management. Given that limited research has dedicated to assessing the impact of quarantine policies, further investigations comparing patient outcomes before and after implementation of such policies are imperative.

Some limitations should be noted for our study. First, the retrospective design itself and the collection of information that relied on medical chart reviews might potentially result in a bias. Second, the small number of study subjects might have resulted in a reduced statistical power of the analysis, mainly for multivariable analysis, which might have underestimated effect sizes or failed to detect significant associations for other quality indicators besides DIT. Third, the study’s single-center nature limits generalizability of our results. Lastly, our analysis did not apply adjustments for multiple hypothesis testing, which could increase the risk of type I error. However, we chose not to adjust for multiple comparisons to avoid inflating type II error, which might obscure clinically meaningful associations. This decision aligns with established literature arguing against routine adjustments in similar contexts (38, 39). Despite this, cautious interpretation of *p*-values is recommended to ensure the robustness of our conclusions. Although the study has the limitations, it has a notable strength in including 3-month mRS scores for a significant portion of the study population. This allowed for an assessment of longer-term functional outcomes and provided valuable insights into the impact of acute stroke management during the COVID-19 era, enhancing the reliability of our findings.

## 5 Conclusion

Our study provides insights into how in-hospital infection control measures can affect the quality of care in hyperacute stroke management. The implementation of stringent quarantine policies impacted DIT and highlighted challenges faced for maintaining efficient stroke care. This emphasizes the need for cautious consideration when adjusting in-hospital quarantine policies for conditions where time-sensitive management is paramount.

## Data Availability

The original contributions presented in the study are included in the article/Supplementary material, further inquiries can be directed to the corresponding authors.
