# Supplementary material for: Impact of in-hospital COVID-19 quarantine policy changes on quality of acute stroke care: a single center experience
Source: Front Neurol. 2025 Jan 7;15:1488529. doi: 10.3389/fneur.2024.1488529 (PMC11746089; doi:10.3389/fneur.2024.1488529)
Supplement: Supplementary file 1 [file Table_1.docx]

Supplementary Material

# Supplementary Figure

**Supplementary Figure 1.** The number of newly confirmed COVID-19 patients in the nearby regions during the study period


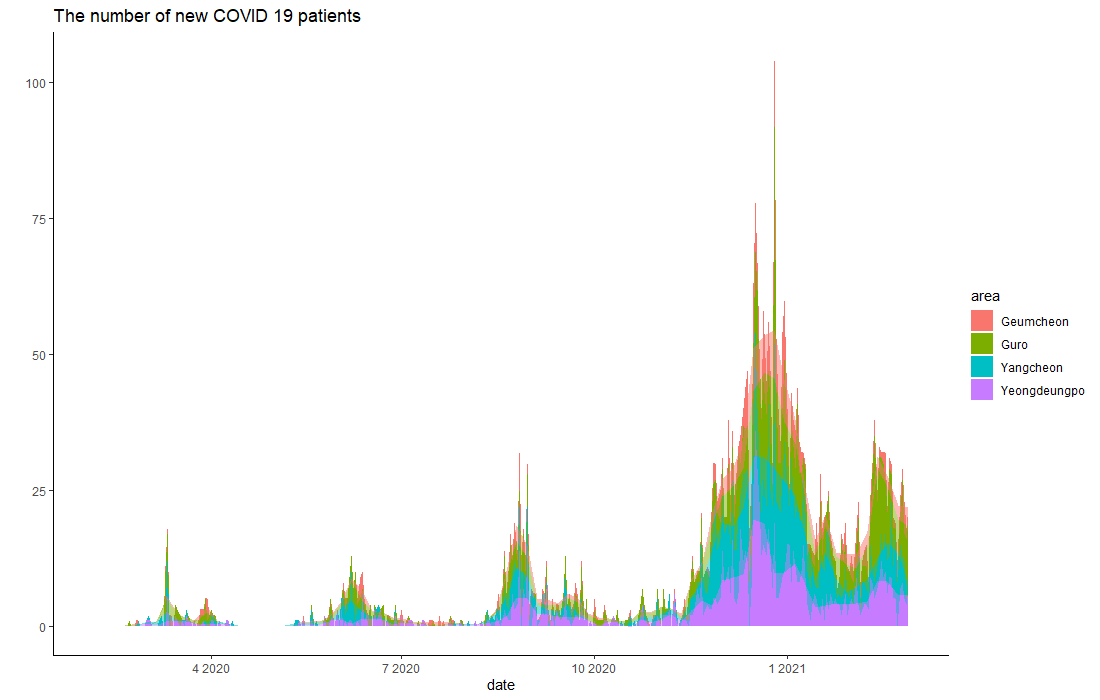


# Supplementary Tables

**Supplementary Table 1.** Quality indicators according to time period

| - Time period | - 2019.1~2019.3 | - 2019.4~2019.6 | - 2019.7~2019.9 | - 2019.10~2019.12 | - 2020.1~2020.3 | - 2020.4~2020.6 | - 2020.7~2020.9 | - 2020.10~2020.12 | - 2021.1~2021.2 | - P for trend |
| --- | --- | --- | --- | --- | --- | --- | --- | --- | --- | --- |
| - Number of patients, N | - 37 | - 31 | - 27 | - 41 | - 39 | - 27 | - 22 | - 24 | - 20 | - 0.032 |
| - Onset to arrival time, min, median (IQR) | - 96 (56-210) | - 65 (35-152.5) | - 108 (41-204.5) | - 95 (31-185) | - 71 (38.5-235) | - 78 (34-147.5) | - 77 (51-236.75) | - 57 (37-176.25) | - 89 (39.25-136.5) | - 0.859 |
| - Door to first image time, min, median (IQR) | - 14 (9-20) | - 9 (7-16) | - 12 (7.5-16.5) | - 10 (7-17) | - 11 (8.5-16) | - 11 (8.5-17) | - 14.5 (10.25-23) | - 19.5 (11-33.5) | - 14 (11.75-21) | - 0.002 |
| - Door to neurologist referral time, min, - median (IQR) | - 19 (10-25) | - 13 (9-23) | - 19 (13.5-30.5) | - 22 (17-31) | - 24 (16-32.5) | - 18 (11.5-27) | - 21.5 (17-35.5) | - 29 (17-37.25) | - 23 (18-34.5) | - <0.001 |
| - Number of intravenous thrombolysis, N (%) | - 4 (10.8) | - 12 (38.7) | - 9 (33.3) | - 16 (39.0) | - 14 (35.9) | - 14 (51.9) | - 6 (27.3) | - 12 (50.0) | - 6 (30.0) | - 0.041 |
| - Door to needle time, min, median (IQR) | - 62.0 (54.75-74.50) | - 47.0 (36.75-53) | - 42.0 (38-56) | - 58.0 (55.75-61) | - 45.5 (37.25-57.5) | - 50.0 (47-61.5) | - 43.0 (38.5-52.75) | - 50.5 (46.5-59) | - 50.5 (48-52) | - 0.769 |
| - Number of endovascular thrombectomy, N (%) | - 8 (21.6) | - 3 (9.7) | - 1 (3.7) | - 7 (17.1) | - 12 (30.8) | - 12 (44.4) | - 6 (27.3) | - 4 (16.7) | - 5 (25.0) | - 0.066 |
| - Door to puncture time, min, - median (IQR) | - 121.5 (111.5-164.75) | - 212 (174.5-224.5) | - 137 (137-137) | - 122 (121-182) | - 137 (131.25-151) | - 145 (135-160.75) | - 169.5 (127.75-296) | - 163.5 (129.75-195.75) | - 229 (190-255) | - 0.014 |
| Discharge mRS 0-2 (%) | - 35.1 | - 45.2 | - 40.7 | - 43.9 | - 25.6 | - 33.3 | - 40.9 | - 45.8 | - 45.0 | - 0.766 |
| - 3-month mRS 0-2 (%) | - 56.8 | - 76.7 | - 55.6 | - 55.3 | - 47.2 | - 50.0 | - 57.1 | - 54.2 | - 65.0 | - 0.549 |

IQR, interquartile range; mRS, modified Rankin’s scale

**Supplementary Table 2.** Comparison of characteristics of patients before and after quarantine in-hospital quarantine policy change

| Variables | Before mandatory COVID-19 screening (N=173) | After mandatory COVID-19 screening (N=95) | P-value |
| --- | --- | --- | --- |
| Age, mean±SD | 69.0±12.8 | 67.8±11.4 | 0.45 |
| Sex, male (%) | 119 (68.9) | 60 (63.2) | 0.86 |
| Onset to arrival time, min, median, IQR) | 89 (36-260) | 77 (39-174) | 0.70 |
| Premorbid mRS, median (IQR) | 0 (0-0) | 0(0-1) | <0.01 |
| Initial NIHSS score, median (IQR) | 5.0(3-13) | 5.0(4-11) | 0.63 |
| Comorbidities, N (%) | | | |
| Hypertension | 105 (60.7) | 57 (60) | 1.00 |
| Diabetes | 48 (27.8) | 29 (30.5) | 0.73 |
| Hyperlipidemia | 21 (12.1) | 12 (12.6) | 1.00 |
| Atrial fibrillation | 23 (13.3) | 11 (11.6) | 0.83 |
| Cancer | 19 (11.0) | 9 (9.5) | 0.85 |
| Smoking (%) | 31 (17.9) | 8 (8.4) | 0.054 |
| Coronary heart disease (%) | 11 (6.4) | 8 (8.4) | 0.70 |
| Stroke or TIA (%) | 31 (17.9) | 18 (19.0) | 0.60 |
| Stroke subtype, N (%) | | | |
| Large artery atherosclerosis | 59 (34.1) | 25 (26.3) | 0.24 |
| Small vessel occlusion | 31 (17.9) | 20 (21.1) | 0.64 |
| Cardioembolism | 45 (26.0) | 27 (28.4) | 0.78 |
| Other-determined | 12 (6.9) | 3 (3.2) | 0.31 |
| Undetermined | 26 (12.0) | 20 (21.1) | 0.51 |
| Large artery occlusion, N (%) | 56 (32.4) | 39 (41.1) | 0.20 |
| Hyperacute reperfusion treatment, N (%) | | | |
| Intravenous thrombolysis | 55 (31.8) | 38 (40.0) | 0.22 |
| Endovascular thrombectomy | 27 (15.6) | 27 (28.4) | 0.02 |

SD, standard deviation; mRS, modified Rankin’s scale; NIHSS, National Institute of Health Stroke. Scale; IQR, interquartile range; TIA, transient ischemia attack
